# Does expressive writing or an instructional intervention reduce the impacts of test anxiety in a college classroom?

**DOI:** 10.1186/s41235-021-00309-x

**Published:** 2021-06-10

**Authors:** Sarah J. Myers, Sara D. Davis, Jason C. K. Chan

**Affiliations:** 1 Department of Psychology, Colorado State University, 410 W. Pitkin St., Fort Collins, CO 80523 USA; 2 University of North Florida, Jacksonville, USA; 3 Iowa State University, Ames, USA

**Keywords:** Test anxiety, Instructional intervention, Expressive writing

## Abstract

Test anxiety is a major concern in education because it causes uncomfortable feelings in test-anxious students and may reduce the validity of exam scores as a measure of learning. As such, brief and cost-effective interventions are necessary to minimize the negative impact of test anxiety on students’ academic performance. In the present experiment, we examine two such interventions: expressive writing (Experiment 1) and an instructional intervention (Experiment 2), with the latter developed from a similar intervention for stereotype threat. Across four authentic exams in a psychology class, students alternated between completing the intervention and a control task immediately before completing the exams. Neither intervention was effective at reducing test anxiety or improving exam performance. The present results suggest that these interventions may not be successful in addressing the impacts of test anxiety in all classroom settings.

**Supplementary Information:**

The online version contains supplementary material available at 10.1186/s41235-021-00309-x.

## Introduction

As tests continue to be a fundamental aspect of higher education, test anxiety remains a critical concern for both students and educators (Zeidner, 1998, 2007). Test anxiety refers to negative thoughts, emotions and bodily symptoms triggered by evaluative situations such as quizzes and exams. The problem of test anxiety is twofold: not only does test anxiety produce uncomfortable reactions in students, it can also reduce their test performance, thereby leading to an underestimation of these students’ academic aptitude. Thus, test results might not be a valid measure of ability for test-anxious students, making identification of interventions to reduce the impact of test anxiety on performance of paramount importance. Unfortunately, treatment options for test anxiety are still limited. Most effective interventions require long treatment plans and a substantial amount of effort and financial resources from students and healthcare providers. The primary goal of the present study was to examine the extent to which expressive writing and knowledge acquisition through an instructional intervention—two brief and cost-effective interventions that have demonstrated promise in laboratory settings—might reduce the negative impact of test anxiety on exam performance in an authentic upper-level college class.

### Effects of test anxiety in education

A significant proportion (15–40%) of students report experiencing test anxiety (Cizek & Burg, 2006; Hill & Wigfield, 1984; McDonald, 2001; Spielberger, Anton, & Bedell, 1976; Zeidner, 1998). Test-anxious students report a range of severe negative thoughts and physiological reactions in evaluative situations (Zeidner, 1998). Beyond their psychological well-being, test anxiety also harms students’ performance during evaluative situations. Indeed, test-anxious students have lower scores on standardized intelligence and aptitude tests (Alpert & Haber, 1960) and lower GPAs (Chapell et al., 2005), even though they spend more time studying compared to their non-anxious counterparts (Culler & Hollahan, 1980; Hembree, 1988). Most importantly, test-anxious students often *underperform* relative to their true ability, as evidenced by the finding that students with high-test anxiety perform as well as or even better than those with low-test anxiety when the pressure of an evaluative situation is alleviated (Beilock, 2008; Deffenbacher, 1978; Ganzer, 1968; Hancock, 2001; Sarason, 1972, 1973). For example, Deffenbacher (1978) asked students with high- and low-test anxiety to solve difficult anagrams. Before the task, students received either high-stress instructions, which emphasized that their performance was a measure of intelligence, or low-stress instructions, which emphasized that this was a difficult task and they were not expected to perform perfectly. Students with high-test anxiety only performed worse than those with low-test anxiety under high-stress instructions, but not low-stress instructions. Consequently, test scores may have poor construct validity as a measure of student abilities because they tend to underestimate the true academic abilities of test-anxious students (Bonaccio, Reeve, & Winford, 2012; Meijer, 2001; Rocklin & Thompson, 1985; Spielberger, 1966; Zeidner, 1990, 1998). Nevertheless, many life-altering decisions such as college admission, scholarships and career opportunities are still influenced by test scores, to the detriment of test-anxious individuals.

Despite its serious implications for the fairness of exams, test anxiety is rarely addressed in student orientations or curricula. Instead, students must seek their own accommodations and treatment if they suffer from test anxiety. Although treatment options exist (e.g., cognitive-behavioral therapy, study skills training, systematic desensitization), they often require substantial time and financial commitments, and they are not always effective (see Ergene, 2003; Von Der Embse, Barterian, & Segool, 2013, for reviews). With the high prevalence of test anxiety and difficulty in receiving long-term mental health treatments, a more realistic solution may be to administer short-term interventions to large groups of students aimed at allowing test-anxious students to perform to their true abilities on exams (i.e., reducing the negative effects of test anxiety on performance).

A thorough understanding of the components of test anxiety may allow such interventions to be developed. Liebert and Morris (1967) first proposed that test anxiety is composed of two main factors, worry and emotionality, and these factors have since received considerable support in the literature (e.g., Chapell et al., 2005; Sarason, 1974). Worry—the cognitive component—refers to intrusive thoughts that individuals experience during tests, such as the consequences of failure or concern about others’ performance (Zeidner, 1998). Emotionality—the physiological component—refers to the bodily changes that students experience in response to evaluative situations, such as an upset stomach or racing heart (Zeidner, 1998). Although test-anxious students report experiencing both components, research suggests that worry affects exam performance more than emotionality (e.g., Chapell et al., 2005; Hembree, 1988) because worrying thoughts occupy working memory resources. Consequently, those worrying thoughts might interfere with memory retrieval and other cognitive operations needed to perform well on exams (Culler & Holahan, 1980; Eysenck & Calvo, 1992; Eysenck, Derakshan, Santos, & Calvo, 2007; Sarason, 1980; Unsworth & Engle, 2007).

This working memory account has received broad empirical support. For example, test anxiety is more detrimental to students with lower working memory capacity (WMC) than students with higher WMC (Tse & Pu, 2012; see also Ansari & Derakshan, 2011a, 2011b; Calvo & Eysenck, 1998; Moran, 2016; Owens, Stevenson, Hadwin, & Norgate, 2014).^1^ The idea is that students with higher WMC can use their more plentiful cognitive resources to buffer against the worrying thoughts that occupy their working memory. Based on these effects of test anxiety on cognitive resources, interventions aimed at alleviating intrusive thoughts should help relieve students from their negative impact. The current experiments examined the effectiveness of two such interventions.

### Expressive writing

One intervention that has been proposed to reduce worrying thoughts is expressive writing, where writers freely write about their concerns, feelings or experiences associated with an undesirable situation (e.g., taking an exam in the present context). Expressive writing has been shown to reduce general anxiety (Alparone, Pagliaro, & Rizzo, 2015; Hines, Brown, & Myran, 2016; Smyth & Pennebaker, 2008; Van Emmerik, Kamphuis, & Emmelkamp, 2008), depression (Frattaroli, Thomas, & Lyubomirsky, 2011; Lepore, 1997) and ruminative thoughts (Gortner, Rude, & Pennebaker, 2006). Expressive writing has also been used successfully among college students. Specifically, asking students to write about their college concerns for several days led to an increase of their GPA the following semester (Lumley, & Provenzano, 2003; Pennebaker & Francis, 1996). These expressive-writing interventions are typically administered for long durations, with participants sometimes writing for multiple sessions across weeks or months. These longer interventions are thought to allow writers to better unpack and understand stressful experiences, eventually leading to changes in their thought patterns surrounding uncomfortable feelings (Pennebaker & Seagal, 1999).

However, some evidence suggests that even one short period of expressive writing can have positive effects on writers. With these short interventions, expressive writing is thought to allow an anxious writer to offload their worries onto paper, so that these worrying thoughts will no longer consume their working memory resources during an upcoming task (Schroder, Moran, & Moser, 2018). These benefits hold particular promise as an intervention for test anxiety because the negative impacts of test anxiety are thought to be driven by worrying thoughts consuming test-takers’ working memory resources (Alparone et al., 2015; Joormann & Tran, 2009; Kellogg, Mertz, & Morgan, 2010; Klein & Boals, 2001; Smyth & Pennebaker, 2008).

One such intervention was used in a study by Ramirez and Beilock (2011), which showed that a brief expressive-writing exercise reduced the negative effects of test anxiety on test performance in high school and college students. This study was especially consequential because the intervention was much shorter than what has been used in previous expressive-writing interventions (on the order of minutes instead of days). In both laboratory and classroom settings, having students expressively write for only 10 min before a test improved test-anxious students’ performance, despite no changes to their level of test anxiety. Ramirez and Beilock (2011) argued that more intensive treatments may be required to reduce test anxiety, but expressive writing may *break the link* between test anxiety and lowered exam performance by freeing up cognitive resources. In other words, students may still have experienced test anxiety, but that experience no longer negatively impacted their performance.

Ramirez and Beilock’s (2011) expressive-writing intervention provides a promising option for a fast-acting, in-class intervention that could allow students’ exam scores to more closely reflect their true ability. However, a recent, widely-cited replication project by Camerer and others (2018, see also Buttrick et al., 2016) cast doubt on the reliability of this finding. Camerer et al. (2018) attempted twice to replicate Ramirez and Beilock’s study (Experiment 2), but both of their amply-powered experiments failed to find a benefit of expressive writing on test performance, with the resulting effect sizes being slightly negative (i.e., expressive writing non-significantly harmed performance). Indeed, closer scrutiny of other studies exploring the effects of expressive writing on test anxiety reveals that results are quite mixed. Some studies have found that expressive writing reduces test anxiety or at least improves test-anxious students’ performance (Allen, 2017; Clinton & Meester, 2019; Frattaroli et al., 2011; Harris et al., 2019; Rozek, Ramirez, Fine, & Beilock, 2019; Shen, Yang, Zhang, & Zhang, 2018; see Park, Ramirez, & Beilock, 2014 for math anxiety). However, a number of studies have not found evidence of these benefits (Allen, 2017; Blank-Spadoni, 2013; Ganley, Conlon, McGraw, Barroso, & Geer, 2021; Relojo-Howell & Stoyanova, 2019; Sefton, 2014; Spielberger, 2015; Walter, 2018; see also Wolitzky-Taylor & Telch, 2010). As one example, Spielberger (2015) tested 110 college students from six undergraduate psychology courses. Prior to a course exam, students either expressively wrote about their anxiety or wrote about what they did the previous day. Spielberger (2015) found that expressive writing did not improve participants’ exam performance compared to their previous course exam score. We were unable to identify any obvious systematic differences among existing studies that would reconcile these contradictory findings. Consequently, our approach is to examine whether expressive writing impacts feelings of test anxiety or performance on authentic college course exams in a high-powered study in Experiment 1.

### Instructional intervention

In addition to the expressive-writing intervention, we also examined the efficacy of a novel instructional intervention. This intervention was developed from a similar intervention used to reduce stereotype threat effects (Johns, Schmader, & Martens, 2005).^2^ Stereotype threat refers to the activation of a negative stereotype about one’s social group or identity, which then causes one to underperform on a task (for reviews, see Appel & Kronberger, 2012; Steele, 1998; Wheeler & Petty, 2001; but see Flore & Wicherts, 2015). For example, a common stereotype is that women perform worse in math than men. When this stereotype is brought to women’s attention before they complete a math task, their math performance is reduced compared to women who are not made aware of the stereotype. This is thought to occur because the added stress of needing to refute the stereotype co-opts cognitive resources, thus leading to lower performance (e.g., Spencer, Steele, & Quinn, 1999; but see Flore & Wicherts, 2015; Stoet & Geary, 2012; Stricker & Ward, 2004).

In their instructional intervention against stereotype threat, Johns et al. (2005) gave men and women a brief essay explaining the impact of stereotype threat on performance, particularly focusing on the fact that lower performance in the face of stereotype threat is not an indicator of one’s true ability (see also Good, Aronson, & Inzlicht, 2003). Remarkably, simply giving students this knowledge allowed women to perform at a similar level as men on difficult math problems (materials for which women tend to perform worse than men under conditions that induce stereotype threat). The researchers theorized that the intervention provided students with an opportunity to reappraise the evaluative situation by attributing their negative thoughts and arousal to an external source (e.g., social pressure) instead of their own perceived incapabilities (see Ben-Zeev, Fein, & Inzlicht, 2005; McGlone & Aronson, 2007). Although Johns and associates’ results were promising, Tomasetto and Appoloni (2013) subsequently found that merely informing students about stereotype threat may not be enough to benefit students’ performance and may even harm performance. Instead, they argued that knowledge of stereotype threat needs to be combined with messages about how to address stereotype threat—a technique that we exercised in Experiment 2.

Although test anxiety and stereotype threat are different psychological constructs, both have been theorized to affect cognition through interfering thoughts and physiological arousal that deplete cognitive resources (Johns, Inzlicht, & Schmader, 2008; Schmader, Johns, & Forbes, 2008; see also Beilock & Ramirez, 2011). Therefore, if learning about stereotype threat allows one to externalize negative ruminations and free cognitive resources, then learning about the nature of and how to address test anxiety may have a similar effect for test-anxious students. Accordingly, we developed an instructional intervention that aimed to teach students about test anxiety, emphasizing the prevalence of test anxiety, the cause-and-effect relationship between anxiety and performance, and coping methods for test anxiety. We tested this intervention as a means to reduce the effects of test anxiety for college students in Experiment 2.

### The current experiments

The two interventions, expressive writing and the instructional intervention, were administered in an upper-level cognitive psychology course at a large public university. As such, our participants were students taking authentic college exams with real stakes in a challenging course. Experiment 1 reports the efficacy of expressive writing for students who enrolled in the course in the fall of 2014, spring of 2015 and spring of 2016, whereas Experiment 2 reports the efficacy of the instructional intervention for students who enrolled between fall 2016 and spring 2017. A rigorous experimental design was used where students alternated between the intervention task and a control task for each of four exams. Thus, an A-B-A-B design was implemented, with the order of tasks counterbalanced across students within each class. For example, half of the students completed the expressive-writing task for Exam X and the control-writing task for Exam X + 1, with the reverse occurring for the remaining students. This design allows within-subject comparisons of exam scores after completing the intervention versus control task—which is statistically more powerful than between-subjects designs that have been used predominantly in the extant literature.

## Experiment 1

In the first experiment, we sought to determine whether completing a brief expressive-writing exercise could reduce the negative effects of test anxiety on exam performance. Immediately prior to each of four course exams, students in a psychology course completed either an expressive-writing or a control-writing task. Note that neither the instructor nor the assigned textbook covered the topic of test anxiety during the semester, except when students were introduced to the experiment during the first class.

## Method

### Participants

Over three semesters, 195 undergraduate students who were enrolled in a 300-level cognitive psychology course at Iowa State University participated in the study. Twenty-two students were removed from data analysis: eight dropped the course, six took the exams in a different location so could not be monitored during the intervention tasks, seven did not complete Exams 2 and 3 (which were used in analyses), and one was retaking the course. The final analyses were completed using the 173 remaining participants. We did not conduct an a-priori power analysis to determine sample size, but based on the effect size reported in Ramirez and Beilock (2011, Experiment 1, *d* = 0.57),^3^ our sample size would provide 0.999 power in a repeated-measures design. Demographic information was not collected.

### Materials and procedure

On the first day of the course, the instructor introduced the research project on interventions for test anxiety. After this brief introduction, the instructor left the room and the experimenter gave all students more details about the project. Students were told they could choose to participate in the experiment in exchange for extra credit,^4^ and those wishing to participate completed a consent form. Next, each student received a paper packet with the alpha span task (Craik, 1986), achievement goals survey (Elliot & Murayama, 2008), backward digit span task (Woodworth, 1938) and trait test anxiety inventory (T-TAI; Spielberger, 1980). All students completed these tasks at the same time in the classroom, with written or verbal instructions given by the experimenter.

The alpha-span task consisted of 14 lists of one-syllable words (Craik, 1986). The students’ task was to listen to the words of each list (e.g., “gulf, mud, corn”) and then recall them in alphabetical order (e.g., “corn, gulf, mud”). List length increased from two to eight words, with two lists for each length. The experimenter described this task and then read the lists one at a time at a rate of about 1 s per word. Immediately after each list was presented, students were given 5 s per word to recall each list. Words that sounded similar to the correct word were considered correct (e.g., “golf” instead of “gulf”) due to the nature of the oral presentation. Minor spelling errors were also accepted. Students’ total alpha span scores corresponded to the total number of lists for which a student recalled all of the presented words in the correct order, with no intrusions.

Following the alpha span task, students completed the achievement goals survey (Elliot & Murayama, 2008). Students read a series of statements (e.g., “My aim is to completely master the material presented in this class.”) and indicated their level of agreement on a five-point scale from “strongly disagree” to “strongly agree.” Scores were calculated as the sum of students’ responses to all survey questions, with a higher score indicating the student had higher motivation to achieve in the course.

Next, students completed the backward digit span task. In this task, students heard 16 lists of digits, one list at a time, and then recalled the digits of each list in the reversed order of how they were presented (Woodworth, 1938). For example, if the experimenter read 2, 5, 3, students should recall 3, 5, 2. List length increased from two to nine digits, with two lists of each length. Students were explicitly discouraged from cheating by recalling the digits in the forward order but simply writing from right to left. Students were given four seconds per digit during recall. Students’ backward digit span score corresponded to the total number of lists for which they recalled the correct digits in the correct order. In hindsight, given that we could not verify whether students cheated on this task, we opted to omit this task from analyses.

Lastly, students’ test anxiety was measured using the T-TAI (Spielberger, 1980). They read statements regarding test anxiety (e.g., “While taking examinations I have an uneasy, upset feeling”) and indicated how often they agreed with the statement on a 4-point scale (1 = almost never, 4 = almost always). T-TAI total, worry and emotionality scores were calculated following scoring methods detailed in Spielberger (1980). After students completed all four measures (alpha span, achievement goals survey, backward digit span and the T-TAI), their packets were collected, the experimenter left the classroom and the instructor returned.

**Fig. 1:**
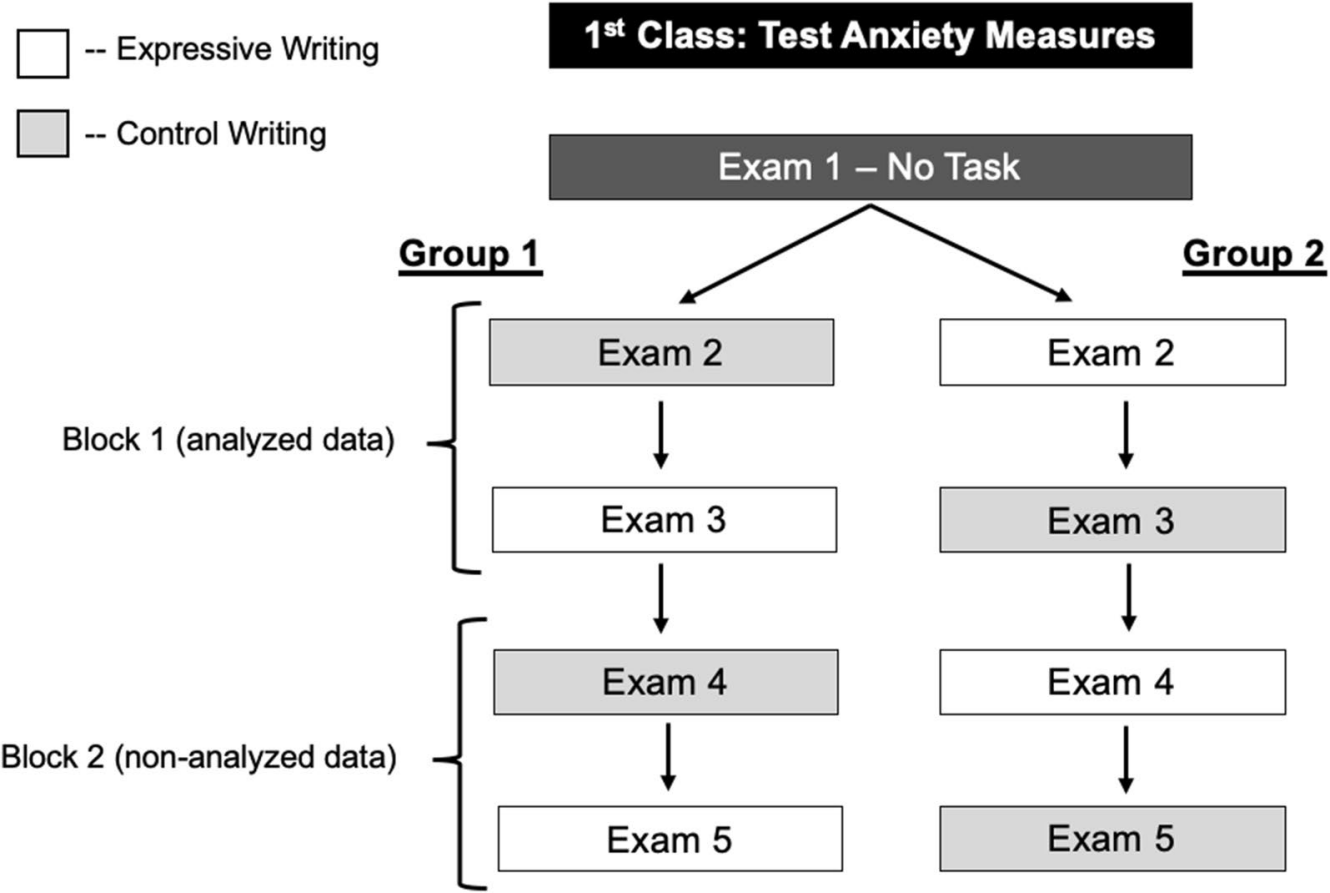
Diagram of procedure used in Experiment 1. After Exam 1, students were randomly assigned to either Group 1 (who completed the control-writing task for Exam 2) or Group 2 (who completed expressive writing for Exam 2). Each group then alternated tasks for the following three exams

Approximately 2 weeks later, students completed their first course exam without any experimental tasks. Exams 2–5 then served as the experimental blocks, and the instructor was not present for these exams. Immediately prior to Exams 2 through 5, participating students completed either the expressive-writing intervention task (Ramirez & Beilock, 2011) or the control-writing task in an A-B-A-B design (see Fig. 1). Students were randomly assigned to complete either expressive writing or control writing for Exam 2, and alternated thereafter so that each student completed the intervention task twice and control task twice. Students not participating in the study sat quietly while participants completed these tasks.

Students were provided with either an intervention task or control task packet when they arrived for their exam. The packets included instructions for the task, blank lines for students to write their responses, and the State-Test Anxiety Inventory (S-TAI; Spielberger, 1980). Our instructions for the writing tasks mirrored those in Ramirez and Beilock (2011). In the *expressive-writing* task, students were given the following instructions:

We would like you to take the next eight minutes to write as openly as possible about your thoughts and feelings regarding the exam you are about to take. In your writing, I want you to really let yourself go and explore your emotions and thoughts as you are getting ready to start the exam. You might relate your current thoughts to the way you have felt during other similar situations at school or in other situations in your life. Please try to be as open as possible as you write about your thoughts at this time.

In the *control-writing* task, students were given the following instructions:

We would like you to take the next eight minutes to write about how you spent your day yesterday. Describe how you spent your time as factually and unemotionally as possible.

All students were encouraged to write for the entire eight minutes. After the writing task, students completed the S-TAI, which asked students to indicate how well each of 27 statements regarding test anxiety described them on a four-point scale (1 = Not at all typical of me, 4 = Very typical of me). A total S-TAI score was calculated for each course exam by summing students’ responses.^5^ S-TAIs were not completed for Exams 4 and 5 because relationships between state test anxiety and writing tasks were established in prior exams. We used both the T-TAI and S-TAI because test anxiety can be measured as both a trait and state (Zeidner, 1998). Trait test anxiety is a student’s overall tendency to experience test anxiety during evaluative situations. In contrast, state test anxiety is a student’s feelings of test anxiety for a specific exam.

Once students finished their pre-exam tasks, they completed the exams at their own pace during the remainder of the class (65 min). All exams used the same format, with 15 multiple-choice questions and three essay questions (they chose two to answer). When a student finished the exam, they turned in both the exam and the pre-exam task packet.

## Results and discussion

For the focal analyses, we report the *p*-value, a standardized effect size measure (Pearson’s *r* or Cohen’s *d*), and the Bayes factor (*BF*). Bayes factors provide a ratio of the likelihood of the data given the alternative hypothesis (i.e., a difference between comparison groups) relative to the null hypothesis (i.e., no difference), expressed as *BF*_10_ (see Kruschke, 2013, for a discussion of Bayes factors). A Bayes factor of 1 means that the data are equally likely under the alternative and null hypotheses. Unlike null hypothesis significance testing, Bayes factors can indicate that the null hypothesis is more probable than the alternative hypothesis (i.e., when *BF*_10_ < 1). For results supporting the null hypothesis, we report the Bayes factors via the reciprocal ratio, denoted as *BF*_01_, such that a larger number provides more support for the null. In other words, a larger Bayes Factor always provides more support of the effect’s direction. Following Rouder, Speckman, Sun, Morey, and Iverson (2009), we used the JZS prior because it requires the fewest prior assumptions about the range of the true effect size. A correlation matrix of the key measures is included in the supplemental materials (Additional file 1: Table S1) available on OSF.

### Test anxiety characteristics

The average T-TAI scores for our students (*M* = 44.04, *SD* = 12.74) were consistent with established norms (Szafranski, Barrera, & Norton, 2012). The two sub-scales, worry (*M* = 16.34, *SD* = 5.36) and emotionality (*M* = 18.73, *SD* = 5.50), were also similar to established norms. The average S-TAI score was in the middle of the range of possible scores (*M* = 66.19, *SD* = 16.29).

### Writing analysis

The content of the writing tasks (both expressive and control) was analyzed using Linguistic Inquiry and Word Count (LIWC) software (Pennebaker, Booth, & Francis, 2007).^6^ LIWC calculates the number of words in a piece of writing from different categories in the program’s corpus (e.g., anxious words – nervous, tense) as a function of the total number of words in the writing. Consistent with our expectation, participants used far more anxiety-related words when completing the expressive-writing task (*M* = 1.9%, *SD* = 1.41%) compared to the control-writing task (*M* = 0.2%, *SD* = 0.44%), *t*(158) = 14.94, *p* < 0.001, *d* = 1.18, *BF*_10_ = 5.90 × 10^28^. This confirmed that students were correctly writing down their worries about the upcoming exam in the expressive-writing task and were not writing about their anxiety during the control-writing task. Further confirming the validity of the expressive-writing task, higher T-TAI scores were associated with more frequent use of anxiety-related words in the expressive-writing task (*r* = 0.29, *p* < 0.001).

### Working memory and achievement goals

Students’ working memory and achievement goals scores had only slight correlations with students’ exam scores and test anxiety (see Additional file 1: Table S1), and conclusions did not change when considering these variables. Therefore, neither measure will be discussed further. For interested readers, additional analyses with working memory are included in the Additional files.

### Carryover effects

Given the nature of the within-subject design, the impact of the intervention could have carryover effects to subsequent exams (e.g., having done a prior expressive-writing task may reduce its future effectiveness or could lead students to implement the intervention on their own in the subsequent control task). Analyses that evaluate this possibility are reported in the supplemental materials and do not suggest that carryover effects impacted the main conclusions.

### Did expressive writing reduce test anxiety?

A negative correlation was found between students’ exam performance and state test anxiety (S-TAI) scores when students completed the control-writing (*r* = −0.20, *p* = 0.01, *BF*_10_ = 2.91) and expressive-writing task (*r* = −0.20, *p* = 0.01, *BF*_10_ = 3.26). A *t*-test was conducted to compare average S-TAI scores after students completed the expressive-writing task compared to the control-writing task. This test indicated that, overall, students’ S-TAI scores did not differ after completing the expressive-writing or control-writing task, *t*(172) = 0.06, *p* = 0.95, *d* < 0.01, *BF*_01_ = 11.77. Thus, it does not appear that expressive writing reduced students’ feelings of test anxiety.

### Did expressive writing improve exam scores?

Although we did not find effects of expressive writing on test anxiety levels, the expressive-writing session may have still reduced the *impact* of test anxiety on exam scores and allowed test-anxious students to perform to their true potential (Ramirez & Beilock, 2011). This is, in fact, the key question of the present investigation. Average exam scores for the four experimental exams are reported in Table 1. As can be seen, performance did not differ between the expressive writing and control conditions for either Block 1 or 2. To maximize our sample size (11% of students dropped the class or missed one or more exams during Block 2), we constrained our analyses to only Exams 2 and 3—the first time students completed the experimental tasks.^7^ Overall, exam scores did not differ after students completed the expressive-writing (*M* = 73%) or control-writing task (*M* = 74%), *t*(172) = 0.37, *p* = 0.72, *d* = 0.03, *BF*_01_ = 11.04. Even among the participants who showed higher test anxiety (i.e., those in the top-half of the T-TAI score distribution, *N* = 87), expressive writing (*M* = 72%) did not improve exam performance relative to control writing (*M* = 72%), *t*(86) = 0.15, *p* = 0.89, *d* = 0.02, *BF*_01_ = 8.36.

**Table 1:** Average exam scores for the experimental blocks as a percentage. Standard deviations are in parentheses

	Block 1	Block 2
Expressive writing	73% (19%)	76% (17%)
Control writing	74% (19%)	76% (16%)

**Fig. 2:**
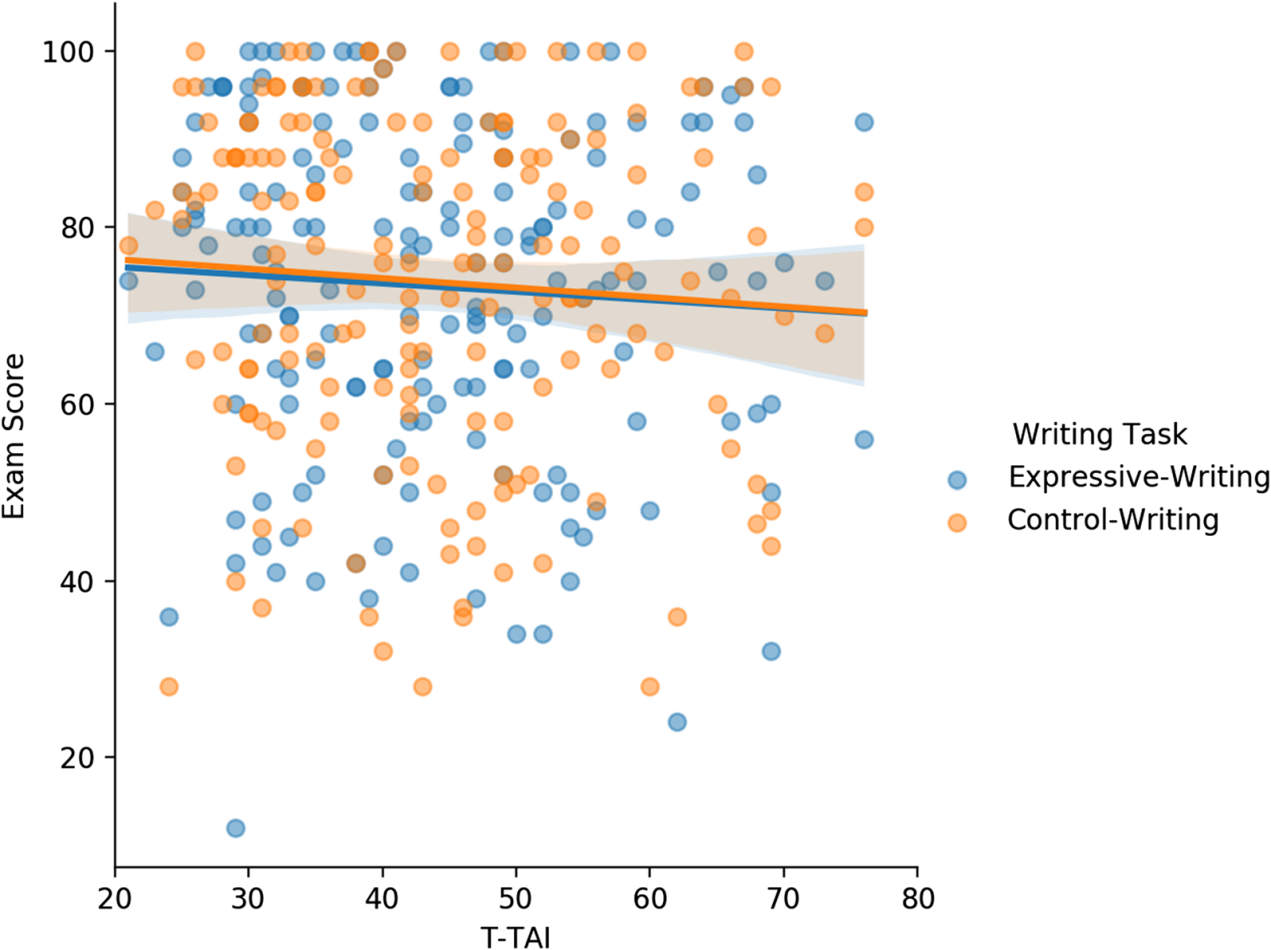
Scatterplot of students’ T-TAI and exam score when they completed the expressive- or control-writing task in Experiment 1. T-TAI scores were collected on the first day of the semester

Next, we considered whether expressive writing changed the relationship between test anxiety and exam scores. The correlation between students’ T-TAI and exam score when they completed the control-writing task was small and non-significant (*r* = −0.07, *p* = 0.35, *BF*_01_ = 6.78), as was the correlation when they completed the expressive-writing task (*r* = −0.06, *p* = 0.41, *BF*_01_ = 7.55). The relationship between T-TAI and exam scores is shown in Fig. 2. As can be seen from the overlapping regression lines, writing task had essentially no effect on the relationship between test anxiety and exam performance. A *z*-test comparing the correlations when students completed the expressive-writing and control-writing task indicated that these correlations did not differ from one another,^8^*z*(two-tailed) =  −0.13, *p* = 0.90.

### Worry sub-component

The T-TAI scale comprises two sub-components: worry and emotionality. Worry has been shown to correlate higher with exam scores than emotionality (Chapell et al., 2005; Hembree, 1988), so we also examined the effects of expressive writing based on students’ worry scores. Including the full sample of students, the correlation between worry and exam scores when students completed the control-writing task was weak but significant (*r* = −0.19, *p* = 0.01, *BF*_10_ = 1.76), as was the correlation when they completed the expressive-writing task (*r* = −0.18, *p* = 0.02, *BF*_10_ = 2.07). Contrary to the idea that expressive writing would weaken the negative effects of test anxiety on exam performance, these correlations did not differ from one another, *z*(two-tailed) = −0.07, *p* = 0.95.

### Strengthening the test anxiety and exam performance relationship

We have reported several analyses above and in the supplemental materials that suggest that expressive writing did not alter the relationship between test anxiety and exam scores. However, one consideration remains: the correlation between test anxiety and exam performance was weak in the present experiment even without expressive writing (i.e., when participants completed the control writing task; *r* = −0.07). It might be difficult for any intervention to decrease such a small relationship. This weak relationship between test anxiety and exam performance was mainly due to many students with low-test anxiety performing poorly on exams and other students with the highest levels of test anxiety performing well on exams (thus not following the expected pattern). To further examine the effectiveness of expressive writing, we conducted an exploratory analysis using a multiverse analysis approach (see Steegen, Tuerlinckx, Gelman, & Vanpaemel, 2016). We selected a subset of participants who exhibited the predicted pattern between test anxiety and exam performance, thereby coercing a stronger negative correlation between test anxiety and exam performance when students completed the control-writing task.

To this end, we separated students using a median split into those with low-test anxiety (T-TAI scores below the median) and high-test anxiety (T-TAI scores above the median). Next, we removed the lowest-performing 25 students in the low-test anxiety group and the highest-performing 26 students in the high-test anxiety group based on their Exam 1 scores, which served as a baseline. This constitutes a removal of 30% of our sample, which is admittedly arbitrary, but we feel that this is a reasonable sacrifice in an exploratory analysis.

**Fig. 3:**
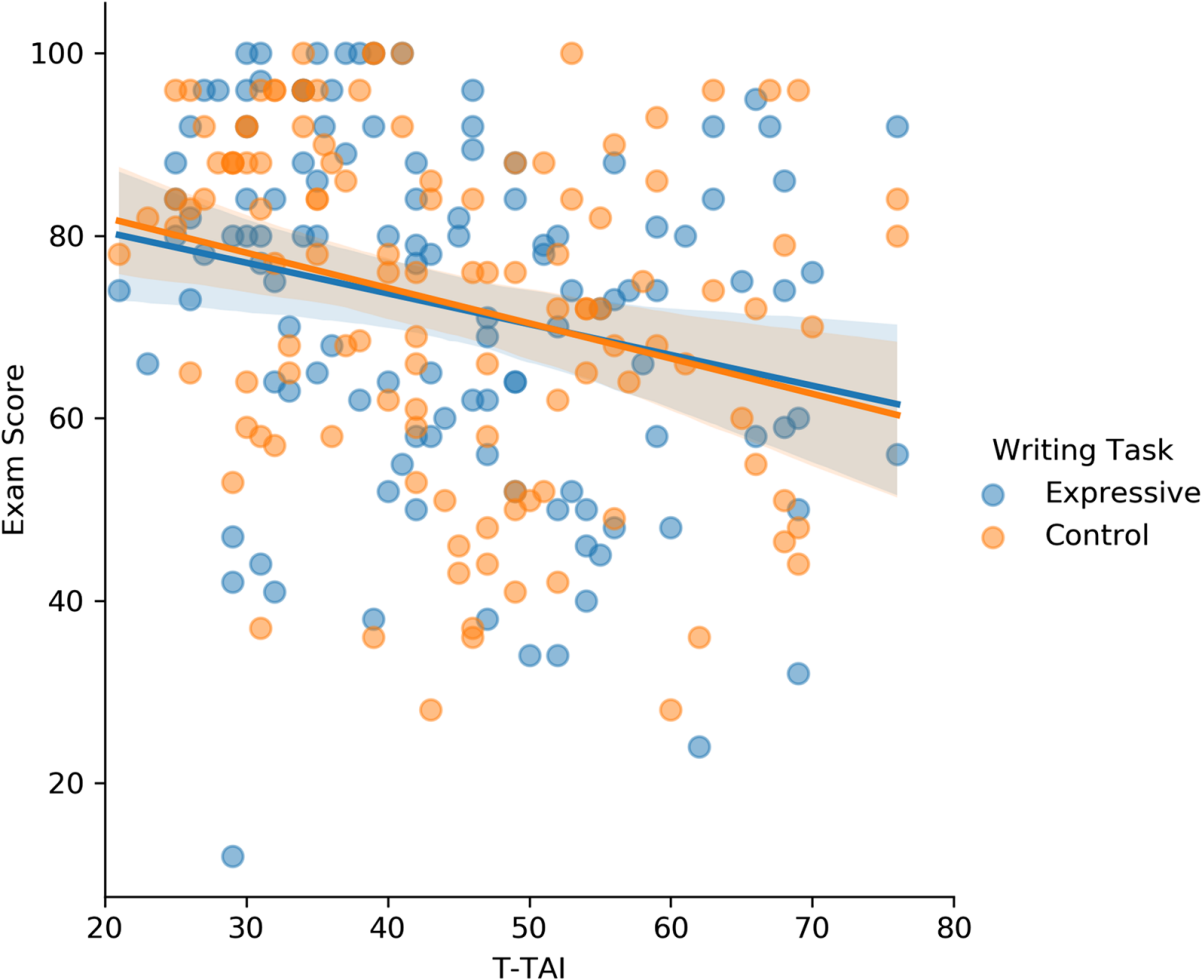
Scatterplot showing a subsample of students’ T-TAI and exam score when they completed the control- or expressive-writing task in Experiment 1. The subsample was created to coerce a stronger negative association between T-TAI and exam scores

This subsample of 120 students produced a strong correlation between students’ T-TAI and exam scores in the “desired” direction (*r* = −0.60, *p* < 0.001, *BF*_10_ = 3.02 × 10^10^) on the baseline exam, thereby establishing a more favorable condition for us to investigate the influence of expressive writing on exam performance. Using this subsample, we examined whether expressive writing diminished the correlation between test anxiety and exam performance for Exams 2 and 3—it did not (as can be seen in Fig. 3). Specifically, participants who completed the control-writing task exhibited roughly the same correlation (*r* = −0.27, *p* = 0.02, *BF*_10_ = 13.70) as those who completed the expressive-writing task (*r* = −0.24, *p* = 0.01, *BF*_10_ = 4.14),^9^*z*(two-tailed) = 0.33, *p* = 0.74. Moreover, across this subsample, expressive writing did not improve participants’ exam performance, *M*_control_ = 73% vs. *M*_expressive_ = 72%, *t*(119) = 0.21, *p* = 0.83, *d* = 0.02, *BF*_01_ = 9.65. In fact, expressive writing did not improve exam performance even when we restricted the comparison to just participants who exhibited high-test anxiety (i.e., the top-half of the distribution in terms of T-TAI) within the subsample, *M*_control_ = 66% vs. *M*_expressive_ = 67%, *t*(59) = 0.47, *p* = 0.64, *d* = 0.06, *B*_01_ = 6.36. Taken together, our results suggest that expressive writing had little to no effects on exam performance regardless of whether there was a strong or weak association between test anxiety and exam performance. In multiverse analyses, it is also important to show that results are not contingent upon the removal criteria used (c.f., Chalkia, Van Oudenhove, & Beckers, 2020a; Chalkia, Van Oudenhove, & Beckers, 2020b). Thus, we also conducted similar analyses after removing 20% and 40% of the sample, and the conclusions remained the same as those presented.

### Experiment 2

Our first experiment showed that expressive writing was ineffective at reducing test anxiety or improving exam scores. In the second experiment, we determined whether an instructional intervention could alleviate the negative effects of test anxiety. Students alternated between reading an essay about test anxiety (instructional essay) and a control essay about an unrelated topic across four exams. Once again, neither the instructor nor the textbook covered the topic of test anxiety.

## Method

### Participants

Over two semesters, 132 students enrolled in a cognitive psychology course participated in the study. Twenty-one students were removed from Experiment 2: nine dropped the course, six were retaking the course, four took the exams in a different location, one worked in the research lab responsible for the project and one chose to stop participating. This left 111 students in the study. Again, we did not perform an a priori power analysis to determine sample size, but based on the effect size reported in Johns et al., (2005, *d* = 1.21),^10^ our sample size would provide 0.997 power in a repeated-measures design.

### Materials and procedure

**Fig. 4:**
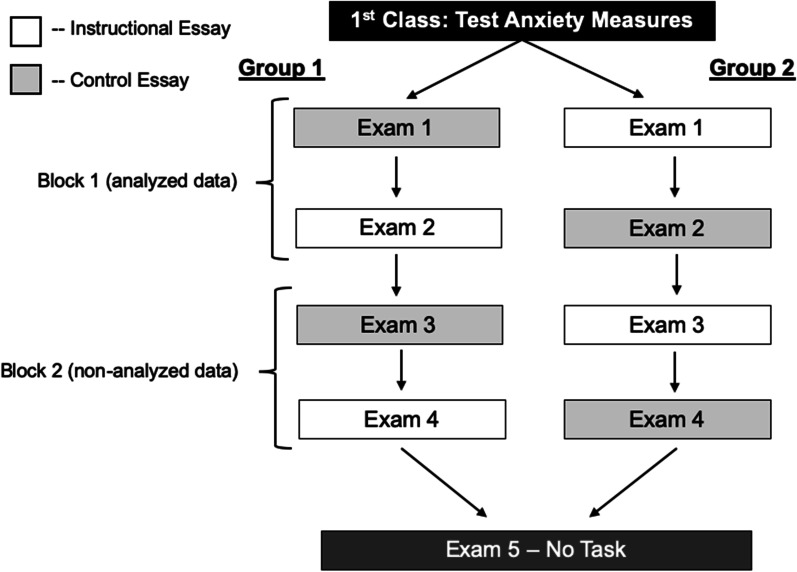
Diagram of procedure used in Experiment 2. Students were randomly assigned to either Group 1 (who read the control essay for Exam 1) or Group 2 (who read the instructional essay for Exam 1). Each group then alternated tasks for the following three exams

The procedure was similar to Experiment 1 but with the following changes that we implemented to increase the likelihood of finding a successful intervention effect. First, we dropped the backward digit span task from the procedure due to the ease of cheating. Second, the S-TAI was administered *after* participants handed in their exams. We opted for this change because we hoped to find a stronger relationship between S-TAI and performance than that observed in Experiment 1, and state test anxiety has a stronger relationship with exam performance when it is administered after students are exposed to the exam than before (Seipp, 1991; Zeidner, 1998). Third, students completed the experimental tasks for Exams 1–4 instead of Exams 2–5 (see Fig. 4 for a diagram)—Exam 1 was not used as a baseline. This was due to the possibility that test anxiety might harm students’ first exam performance more than later exams. Thus, an intervention may be more impactful for the first course exam than for later ones. Fourth and most importantly, participants were given the instructional intervention rather than expressive writing. A different control task was also created for Experiment 2 to be analogous to the instructional intervention. For both tasks, participants read an essay, took a three-question multiple-choice quiz over the essay, and lastly reviewed written feedback of the quiz. The entire task took about 12 min.

The instructional intervention was a three-page essay (985 words) discussing the prevalence of test anxiety, how test anxiety can hinder performance via cognitive interference, and brain regions that are impacted by test anxiety (see Appendix 1). Lastly, the essay covered what students can do about test anxiety, including cognitive behavioral therapy, more frequent testing and expressive writing. Thus, our instructional intervention taught participants the nature of test anxiety (similar to Johns et al., 2005, but at a more extensive scale) and provided them with information on how to address test anxiety (thereby fulfilling the recommendation made by Tomasetto & Appoloni, 2013). We also included a brief quiz to check students’ understanding. For the control task, students read an essay (923 words) about handedness (see Appendix 2), which explained the genetic traits and cultural influences that determine handedness. Thus, we incorporated an *active control condition* that was absent in some prior studies (e.g., Johns et al., 2005).

## Results and discussion

Data were analyzed using the same methods as Experiment 1. A correlation matrix of the key collected measures is included in the supplemental materials (Additional file 1: Table S2).

### Test anxiety characteristics

The average T-TAI for this sample of students (*M* = 46.52, *SD* = 13.05) were again consistent with established norms (Szafranski et al., 2012), as were worry (*M* = 17.08, *SD* = 5.25) and emotionality (*M* = 19.79, *SD* = 5.83) averages. The average S-TAI score was in the middle of the range of possible scores (*M* = 70.42, *SD* = 16.44).

### Working memory, achievement goals and carryover effects

Our conclusions of the study again did not change when considering the variables of WMC and achievement goals, so neither measure will be discussed further. Nonetheless, we reported data concerning working memory and possible carryover effects in the supplemental materials.

### Essay quiz questions

Students answered a majority of the essay quiz questions correctly, although students scored better on the test anxiety quiz (*M* = 2.80, *SD* = 0.40) than the handedness, control quiz (*M* = 2.30, *SD* = 0.70), *t*(110) = 7.21, *p* < 0.001, *d* = 0.69, *BF*_10_ = 1.20 × 10^8^. Note that due to disparities in both the content and the questions themselves, performance differences in the two quizzes are not interpretable. The correlations between students’ test anxiety (T-TAI) and their control essay quiz score (*r* = −0.18, *p* = 0.06, *BF*_01_ = 1.52) and instructional essay quiz score (*r* = −0.19, *p* = 0.04, *BF*_01_ = 1.07) were both near the threshold of statistical significance, suggesting that students’ test anxiety may even impact their performance on a low-stakes quiz. However, we caution against over-interpreting these results given their borderline effects.

### Did the instructional intervention reduce test anxiety?

Negative correlations were found between students’ exam performance and S-TAI scores when they read the control essay (*r* = −0.23, *p* = 0.02, *BF*_10_ = 2.15) and the instructional essay (*r* = *−*0.32, *p* < 0.001, *BF*_10_ = 32.37), and these correlations did not differ from each other, *z*(two-tailed) = 0.94, *p* = 0.35, meaning that higher state test anxiety predicted lower exam performance regardless of the task students completed. Students’ average test anxiety on exam days after completing the instructional (*M* = 70.1) versus control essay (*M* = 70.7) also did not differ, *t*(110) = 0.80, *p* = 0.42, *d* = 0.08, *BF*_01_ = 6.94, suggesting that the instructional intervention did not reduce students’ feeling of test anxiety. However, note that because the S-TAI was administered after students completed the exam in Experiment 2, their S-TAI scores might have also been impacted by their experiences with the exam in addition to any impact of the intervention and control essays.

### Did the instructional intervention improve students’ exam scores?

Average exam scores are reported in Table 2. Again, only data from the first exams for which students completed the instructional and control readings (Exams 1 and 2)^11^ were analyzed. Overall, exam scores did not differ after students read the instructional (*M* = 69%) or control essays (*M* = 70%), *t*(110) = 0.42, *p* = 0.67, *d* = 0.04, *BF*_01_ = 8.71. The instructional intervention did not enhance exam performance even when we limited the analysis to only participants who reported higher test anxiety (i.e., top half of the distribution according to their T-TAI score, *N* = 55), *M*_instructional_ = 65%, *M*_control_ = 69%, *t*(54) = 1.42, *p* = 0.16, *d* = 0.19, *BF*_01_ = 2.63.

**Table 2:** Average exam scores for the experimental blocks as a percentage

	Block 1	Block 2
Instructional intervention	69.2 (20.8)	80.0 (16.2)
Control task	70.0 (19.5)	78.7 (15.9)

Standard deviations are in parentheses

**Fig. 5:**
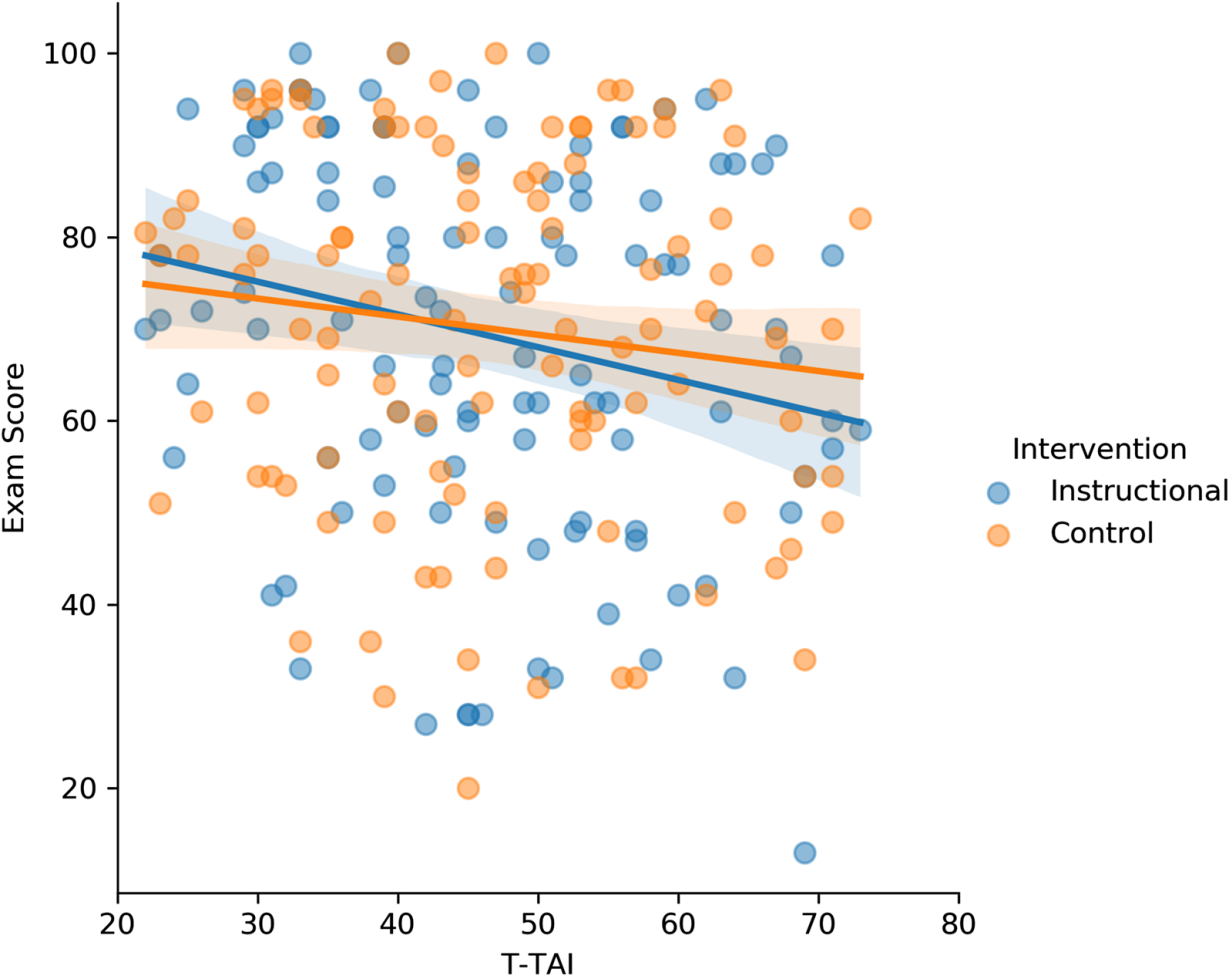
Scatterplot of students’ T-TAI and exam score when they completed the instructional or control task in Experiment 2. T-TAI scores were collected on the first day of the semester

The correlation between students’ T-TAI and exam scores when students read the control essay was numerically stronger than Experiment 1, but still non-significant (*r* = −0.13, *p* = 0.17, *BF*_01_ = 3.32). In contrast to our prediction, students exhibited a stronger negative association between T-TAI and exam score when they read the instructional essay (*r* = −0.22, *p* = 0.02, *BF*_10_ = 1.88). However, these correlations did not significantly differ from each other, *z*(two-tailed) = 1.03, *p* = 0.31. This again suggests that the intervention did not weaken the relationship between test anxiety and exam performance. Indeed, if anything, the instructional intervention exacerbated the association between test anxiety and exam performance. These associations are depicted in Fig. 5.

### Worry sub-component

The correlation between worry scores and exam performance when students read the control essay was moderate and significant (*r* = −0.23, *p* = 0.01, *BF*_10_ = 2.45). Contrary to the hypothesis that the instructional intervention would weaken this relationship, the correlation between worry and exam performance when students read the instructional essay was numerically (*r* = −0.28, *p* = 0.003, *BF*_10_ = 9.83) but not significantly stronger, *z*(two-tailed) = 0.53, *p* = 0.60.

### Strengthening the test anxiety and exam performance relationship

**Fig. 6:**
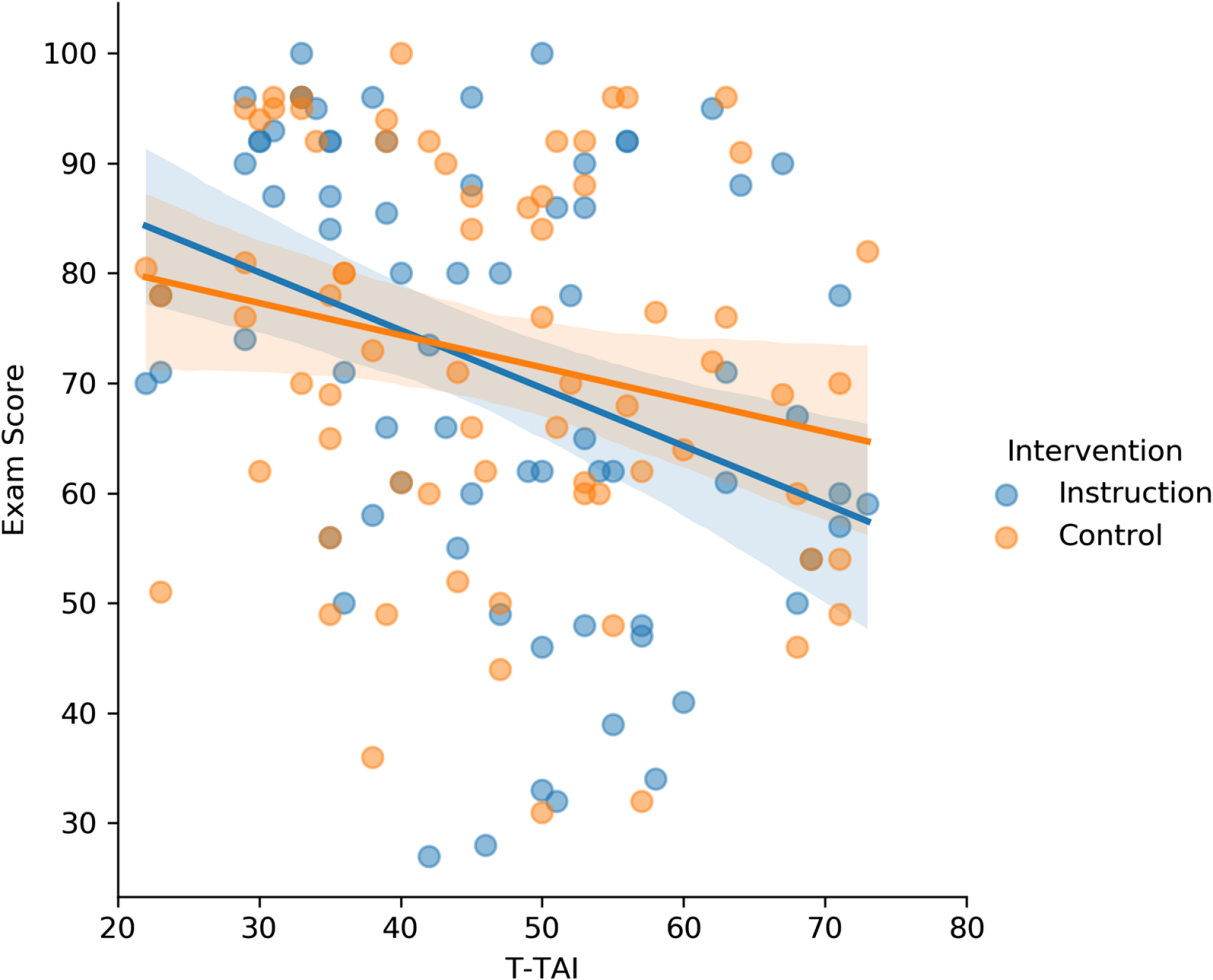
Scatterplot showing a subsample of students’ T-TAI and exam score when they completed the control or instructional task in Experiment 2. The subsample was created to coerce a stronger negative association between T-TAI and exam scores

Although the relationship between test anxiety and exam performance was stronger than in Experiment 1, these correlations were still weak. Thus, we repeated the same procedure as Experiment 1 to select a group of participants who displayed a stronger correlation between T-TAI and exam score. Unlike Experiment 1, we did not administer a baseline exam for which participants did not complete any task beforehand. Therefore, we used participants’ control exam score in Exam 3 or 4 as the baseline performance and to establish its association with participants’ T-TAI score. We again separated students using a median split into those with low- and high-test anxiety and then removed the lowest-performing 15 students (based on their Exam 3 or 4 score) with low-test anxiety and the top-performing 15 students with high-test anxiety. This again constituted a removal of 30% of the sample, and we reached the same conclusions when we removed 20% and 40% of the sample. Nine additional participants did not complete the control Exam 3 or 4 and were removed from this analysis. This left a total of 71 participants in the subsample. This subsample exhibited a robust correlation between students’ T-TAI and exam scores (*r* = −0.61, *p* < 0.001, *BF*_10_ = 1.13 × 10^6^). The results from this subsample echoed that from the full sample, such that students showed a numerically, but not significantly, stronger negative association between test anxiety and exam score when they completed the instructional intervention (*r* = −0.35, *p* = 0.003, *BF*_10_ = 10.99) than when students completed the control task (*r* = −0.22, *p* = 0.07, *BF*_10_ = 0.77), *z*(two-tailed) = 0.81, *p* = 0.42. See Fig. 6 for a scatterplot of these associations. Once again, if anything, the instructional intervention might have exacerbated the association between test anxiety and exam performance in this subsample.

## General discussion

Test anxiety remains a major concern in education given the continued use of testing to inform life-changing decisions. Not only does test anxiety cause unpleasant physiological and psychological reactions in students, it has also been suggested that exams may underestimate the true academic ability of test-anxious students (Deffenbacher, 1978). This study examined two short, inexpensive interventions aimed to reduce these negative effects of test anxiety: expressive writing and an instructional intervention based on a similar intervention developed for stereotype threat (Johns et al., 2005). The current study did not find convincing support for either intervention. Neither expressive writing nor the instructional intervention reduced feelings of test anxiety (assessed via S-TAI scores) or improved exam scores, with Bayes factors indicating that the null hypothesis (i.e., no difference between scores after completing the intervention and control tasks) was much more likely than the alternative hypothesis. Most importantly, the two interventions did not reduce the detrimental effect of test anxiety on exam performance, even when we selected a sample of students who showed a strong test anxiety–exam score correlation or used the worry sub-component. Our conclusion is that expressive writing and an instructional intervention were not effective at addressing the impact of test anxiety in the present college class.

The main concern with the present study is that the relationship between students’ trait test anxiety and exam scores was weak. This suggests that, on average, students’ exam performance may not have been strongly influenced by their test anxiety in the course. Nevertheless, even though correlations were weak in the present study, they were not much lower than typical correlations between test anxiety and performance reported in large studies (*r* = −0.15 to −0.21; Chapell et al., 2005; Hembree, 1988; Schwarzer, 1990). After all, test anxiety is only one factor impacting students’ exam grades, and that relationship may be more complex than typically expected. Thus, it is important to consider other factors that can moderate the association between test anxiety and exam performance. For example, Culler and Holahan (1980) found that among students with high-test anxiety, better study habits were associated with higher test scores (see also Putwain & Daly, 2013, for impact of academic buoyancy; Rozek et al., 2019, for socioeconomic status).

One may wonder whether the weaker correlation between test anxiety and exam performance relative to some other studies (e.g., Ramirez & Beilock, 2011) was due to students in the cognitive psychology course experiencing lower test anxiety. We do not believe this is the case because our T-TAI scores were similar to other normative scores (Szafranski et al., 2010). Thus, the students in our samples appeared to experience similar levels of both trait- and state-level test anxiety to other large samples of college students with sufficient variability and a relatively normal distribution. We also conducted multiple analyses to determine whether the interventions impacted some students more than others. First, we examined whether our interventions improved exam performance for only participants who reported higher-than-average test anxiety. Second, we conducted analyses using students’ worry scores from the T-TAI subscale, which was more strongly correlated with exam performance than the overall T-TAI score which also includes an emotionality subscale (see Chapell et al., 2005; Hembree, 1988). Third, we determined whether the impacts of the interventions depended on students’ WMC (see supplemental materials). Lastly, we used multiverse analysis logic to conduct exploratory analyses in which we constrained our analysis to only a sample of participants who displayed a strong correlation between test anxiety and exam performance (i.e., higher test anxiety was associated with lower exam scores). All analyses converged on the same conclusion: the interventions (1) did not improve exam performance and (2) did not reduce the test anxiety–exam performance relationship.

It might also be argued that we have merely shown that an expressive-writing intervention and an instructional intervention were ineffective in a college-level cognitive psychology class taught at a large public university. This criticism is certainly justified given that we had only tested our intervention in this setting (although this is an argument that can be leveled at most studies), but it is important to note several positive aspects of the present experiments in terms of both internal and external validity. First, these experiments were conducted in an authentic college classroom, students were taking exams with real stakes, and their measured test anxiety was consistent with established norms. All of these conditions are precisely where educational researchers would want to test these interventions. Second, both of our experiments were well-powered and included much larger sample sizes than some previous studies, particularly in the classroom environment. Third, as we will discuss below, a number of other well-powered studies have also failed to demonstrate an effect with these and similar interventions.

Although our results were dissimilar to Ramirez and Beilock’s (2011), they are consistent with other studies that have also found no benefit of expressive writing for test anxiety (Allen, 2017; Blank-Spadoni, 2013; Camerer et al., 2018; Sefton, 2014; Spielberger, 2015; Walter, 2018). Thus, it appears that the benefits of expressive writing remain mixed, and this intervention might not be effective in all situations. In the present study, the intervention might not have been effective because students were not given enough time to expressively write. However, our time limit of eight minutes for expressive writing was comparable to other studies – Ramirez and Beilock (2011) gave participants ten minutes, and Park et al. (2014) gave participants seven minutes. Therefore, we do not believe that the short writing duration was responsible for our null effect. Nevertheless, there may be other methodological differences between the studies that could account for why expressive writing appears beneficial in some studies but not in others.

We also did not find a benefit for the instructional intervention, in which students read about causes and treatments of test anxiety. This was a novel intervention that had not previously been used to address test anxiety. Although it was developed based on a similar intervention to reduce the effects of stereotype threat (Johns et al., 2005) and both stereotype threat and test anxiety effects have been proposed to occur due to the same cognitive mechanisms, there could be key differences between stereotype threat and test anxiety which caused the interventions to have differing effects. Importantly, some studies indicate that an instructional intervention reduced stereotype threat effects because it allows students to attribute their stress to an external source, specifically social pressure to disprove the stereotype (Ben-Zeev et al., 2005; Johns et al., 2005; McGlone & Aronson, 2007). This social component might be less applicable to test anxiety. Consequently, learning about the causes and effects of test anxiety may ironically lead students to judge themselves for experiencing this anxiety rather than attributing their test anxiety to an outside source. Another possibility raised is that the impacts of the instructional intervention may depend on students’ motivation toward cognitive psychology. Stereotype threat research has shown that those most impacted by stereotype threat are ones who strongly feel that the test domain is important to them (e.g., women who want to do well in math; Spencer et al., 1999; Steele, 1997). Thus, it is possible that not all students in the present study found cognitive psychology important to their own identity. Nevertheless, we believe that the pressure students experienced in this authentic exam environment was likely to be as least as strong, if not stronger, than what participants would have experienced in a laboratory-based environment, given that students in the present study were taking a course that contributes to their college GPA. Moreover, it might be argued that the conditions of our experiments are exactly those that we wish the interventions to have a positive impact.

However, it is also important to note that some studies have not replicated the benefits of the instructional intervention developed by Johns et al. (2005) for reducing stereotype threat (Rivardo, Rhodes, Camaione, & Legg, 2011; see also Fuller, 2013). We could identify no systematic differences between studies that would account for these diverging results. Therefore, it is possible that instructional interventions are also not effective in all circumstances. In a meta-analysis of stereotype threat interventions, Liu, Liu, Wang, and Zhang (2020) found a moderate effect of reappraisal interventions (including the intervention used by Johns et al., 2005), but they noted that the studies were impacted by publication bias and thus may overestimate effect sizes. Most importantly, not all stereotype threat interventions appear to work in every context (e.g., Rivardo et al., 2011).

## Conclusions and practical implications

Based on the present results, we cannot recommend the use of expressive writing or instructional essays as blanket interventions to reduce the effects of test anxiety among all college students. At the very least, the putative benefits of these interventions do not apply in all educational settings, and future research is needed to clarify what factors might moderate the relationship between these interventions, test anxiety and exam performance.

We end with a few recommendations about exams and test anxiety in the classroom. Given the negative effects of test anxiety, educators and policy-makers may argue for the removal of exams from curricula (see e.g., Buck, Ritter, Jensen, & Rose, 2010). However, this would not be the best solution for students; tests are not only a means of assessment but importantly are also a learning tool for students. Indeed, test taking, which requires memory retrieval, is a well-supported method to improve students’ long-term retention of the tested material and to learn new material (for reviews, see Chan, Meissner, & Davis, 2018; Rowland, 2014). More experience with retrieval practice has even been shown to reduce students’ feelings of test anxiety (Agarwal, D’Antonio, Roediger, McDermott, & McDaniel, 2014; see also Yang et al., 2020). Thus, we encourage educators to keep testing students within their classrooms. However, the negative impacts of test anxiety on some students’ performance should be considered when using exam performance to make life-changing decisions. As mentioned previously, exam performance often underestimates test-anxious students’ true ability. Thus, it is essential that decisions are made using a holistic approach of the student rather than any one test score (for other recommendations, see Zeidner, 2007).

### Supplementary Information

**Additional file 1**. Supplementary materials including additional data analyses.

## Data Availability

The datasets used during the current study are available at https://osf.io/x5w8z/?view_only=979ade23442544a4bf0fba26420db517.

## Appendix 1

### Instructional essay and quiz about test anxiety

#### What does science tell us about test anxiety?

Many students in the US suffer from test anxiety. Test anxiety refers to a set of undesirable emotional, mental and physiological responses associated with test taking (Zeidner, 1998). Students with high-test anxiety often perform worse on tests than students with low-test anxiety. An important question is this: Do people become test anxious because they are poor test takers and that they simply cannot do well on exams? If this were the case, then perhaps little can be done for test-anxious individuals, because they simply lack the ability to do well on exams. However, an equally probable, opposite possibility exists: Do people perform poorly on exams because their anxiety disrupts their ability to concentrate? If this were the case, then as long as students are relieved from their anxiety, they can perform just as well as students who do not feel anxious about exams.

Recent research suggests that high anxiety test takers do worse than low anxiety test takers, not because they have less ability, but because the anxiety-related thoughts and feelings interfere with their normal cognitive processes, such as their ability to stay focused during an exam (Zeidner, 1998). In fact, anxious test takers only underperform relative to their potential when they see the test as a high stakes situation. When anxious test takers are told that scores on an exam do not count, they can perform just as well as non-anxious test takers.

#### How does test anxiety affect performance?

Test anxiety isn’t all that different than other forms of anxiety (e.g., performance anxiety for sports, math, or even other forms for fear), except that it arises when test takers feel the pressure to perform and view the test as a high stakes situation. There are several ways in which test anxiety can negatively affect performance. First, it can reduce a student’s motivation to do well, which then reduces how hard a student studies or prepares for the exam. Second, and perhaps more importantly, test anxiety can cause one to ruminate about the possibility of failure, which can serve as a source of distraction (Eysenck et al., 2007). For example, when an anxious test taker encounters what feels like a difficult question, the test taker may think that “I can’t do this,” which then interrupts his/her ability to focus on answering the question. It is important to note that every student will encounter some questions that are more difficult than others, but that non-anxious test takers are just less likely to be distracted by thoughts unrelated to the exam.

From a biological perspective, anxiety is often the response to threats in the environment. Perceived threats can trigger a cascade of responses in the sympathetic nervous system. Perhaps most relevant here is the idea that when a threat is encountered, the amygdala, a structure deep inside the subcortical brain, triggers activities in the hypothalamus to release stress hormones (e.g., cortisol), which can inhibit functioning of the hippocampus (important for memory) and cortical structures important for regulating attention (Kim & Diamond, 2002).

#### What can you do about test anxiety?

Many factors contribute to test anxiety, chief among them are the stress induced by transitioning to a new environment. Many students experience difficulty when moving from high school to college or from entry-level to upper-level college classes. The new environment, larger class size, new classmates and new instructors all contribute to students feeling anxious during exams. Moreover, college students often take exams in a room filled with strangers while being observed by proctors. These circumstances can trigger anxious reactions in students and reduce their concentration.

As has been described earlier, there are parallels between the anxious response triggered by tests and other anxiety-inducing situations. And like other forms for anxiety, test anxiety can be reduced via systematic desensitization and cognitive behavioral therapy (Hembree, 1988). What is particularly intriguing is that students become progressively less test-anxious when exams are scheduled more frequently than less frequently (Agarwal, D'Antonio, & Roediger, 2014). This can happen because of desensitization or that students do not perceive the test situation to be as high stakes (because more exams means fewer points are allotted to each exam).

Recent research has shown that individuals are equipped with the ability to combat the negative influence of test anxiety on performance. For example, asking students to write down their thoughts and feelings about exams on a sheet of paper for 20 min (i.e., expressive writing) can reduce anxiety-based performance deficits (Ramirez & Beilock, 2011). Alternatively, having people reframe their perception of an anxiety-inducing situation (e.g., the stress you feel can actually be beneficial) can improve performance by anxious test takers (Palumbo et al., 2014). One reason these interventions work is because they allow individuals to invoke cognitive control processes that help them reappraise their negative emotional response, thus freeing them from distracting ruminating thoughts. The following figure shows the frontal and parietal brain regions that are involved in combating anxiety-based performance deficits.

In a few minutes, you will be taking a test on what you have learned in the past few weeks of class. If you feel anxious about the exam, it is important to keep in mind that this anxiety could be the result of environmental factors such as transitioning to upper level classes and has nothing to do with your actual ability to do well on the exam. More importantly, you can overcome the negative influence of anxiety on performance simply by reframing your expectations and feelings toward the exam. Remember that this is but one of several exams in the class, and that it accounts for 14% of your final grade. If you feel at all anxious, just know that this is completely normal, and you shouldn’t assume that it means that you are doing poorly on the test.

QUIZ QUESTIONS:

Please answer the following questions based on what you have learned from the article. It is important that you answer the questions in the order as they appear on the page, and **do NOT proceed to the next page of this packet until you have finished answering all the questions** on this page.

1. People with high-test anxiety performs poorly on exams because
  - They are bad test takers, and there is little that can be done to help them
  - They don’t learn information as well as people who do not have test anxiety
  - **Their anxiety interferes with their concentration during exams**
  - They are not as smart as people without test anxiety
2. People with high-test anxiety often get “hung up” on difficult questions because
  - They are poorly prepared for the exam
  - **Their anxiety causes them to ruminate about failure**
  - They are looking for excuses
  - They are not trying hard enough
3. What can be done to reduce the negative impact of test anxiety on performance?
  - Not much can be done
  - Study harder
  - Try not to thinking about failure
  - **Take exams more frequently**

## Appendix 2

### Control essay and quiz about handedness

#### Why are most people right-handed?

The short answer is that you are more likely to be right-handed because most of humanity is right-handed. About 90% of us are righties, although almost all the information we have on this comes from Western countries after the year 1900.

Why are people right-handed? The reality is that we don’t really know what causes handedness at all. In fact, there is no known inherent biological tendency toward people being right-handed. This actually makes the existence of a righty majority confusing and fascinating. Most of us think of handedness as simple and binary. Even scientists used to think the distinction between lefties and righties was mostly genetic. However, that is incorrect.

Here is what we do know: Humans are relatively asymmetrical creatures. That applies to both how we use the body parts we have and where things are in the first place. Our hearts tend to be on the left. Our livers tend to be on the right. Not only do these asymmetries exist, but some of them seem to be interconnected. People who are right-handed tend to process language on the left side of their brain.

There is a way to see this connection: the Wada Test, a tool that doctors sometimes use to prepare people for brain surgery. First, the doctor instructs the patient to hold up both hands and talk. Then an injection of barbiturates is made to the left carotid artery. As soon as the drugs hit the left side of the brain and anesthetize it, 90% of right-handed people will lose control of their right hand and they will become unable to speak. However, about 70% of left-handed people will *also* lose their language ability when the left side of their brain is anesthetized. The rest are about evenly split between processing language on the right and processing it with both sides. Nobody knows why most righties are asymmetrical and most lefties aren’t.

#### What role does nature play?

Scientists used to think that handedness was a simple trait, easily explained. One of the most popular genetic models for handedness was proposed by psychologist Chris McManus (1985). Called the “dextral/chance” model, it proposed that handedness is determined by a single gene that comes in two varieties, dextral (D), meaning “righty” and chance (C), meaning literally just chance. People who got a C variant from both parents would have a 50–50 chance of being lefties. The pairing of CD would take that chance down to 25%. People with DD would all be right-handed.

More recently, however, scientists demonstrated that handedness involves far more genes than that (Armour, Davison, & McManus, 2013). The researchers analyzed the genomes of 3,940 twins whose handedness was recorded from previous studies. First, they found that identical twins, who share all genetic material, were not significantly more likely to have the same dominant hand than fraternal twins. This finding thus rules out simple genetics. Second, the researchers failed to find any genes that stood out as connecting unrelated people who had the same dominant hand. If the genetics of handedness were simple, we should be able to identify the genomes of unrelated righties and discover a gene or genes they all shared.

Despite this, McManus (who is an author on both the 1985 and 2013 studies) claimed that the dextral/chance model is still the best explanation to date. The important thing is that there probably is no single gene that is responsible for left-handedness. However, it is still likely that we are dealing with genes that either make a person right-handed or leave her handedness to chance, the latter of which results in some left-handed people. All that has changed, McManus argued, is that we now know this must involve many genes, rather than just one.

#### What role does nurture play?

Current research shows that genetics only accounts for about 25% of the variation in handedness. To put it in context, blood groups (the immune system categories that determine who can take a blood transfusion from whom) are based on simple inheritance and are almost 100% attributable to genetics. Height is more complex, involving 300-odd genes, the most powerful of which accounts for only about 4 mm of growth, but even that is highly heritable, with scientists claiming that 60–80% of a person’s height being determined by genetics. Body mass index, skin color, hair color, eye color—most obvious physical traits have high rates of genetic heritability. Handedness is a distinct outlier.

Aside from genetics, handedness can be affected by social forces. In most Western cultures, for instance, generations born at the beginning of the twentieth century had left-handedness drilled out of them, says Tulya Kavaklioglu, a graduate student at the Max Planck Institute for Psycholinguistics who is studying the connections between handedness, language and genetics. A study on the prevalence of left-handedness among Australians showed that only about 2% of Australians born in 1880 were left- handed; however, of the generation born in 1969, 13% were lefties. As it became more acceptable to be a lefty, Kavaklioglu says, more people were.

So is it nature or is it nurture? It is difficult to make all this evidence fit together. But researchers agree that it is easier if one approaches heritability as more than genetics. People are right-handed because of genes. People are right-handed because of culture. And people are right-handed because of other factors that affect them both before and after birth—things that can be heritable without being genetic.

This essay is based on an article written by Maggie Koerth-Baker published at fivethirtyeight.com. Wording in the article has been revised.

QUIZ QUESTIONS:

Please answer the following questions based on what you have learned from the article. It is important that you answer the questions in the order as they appear on the page.

1. Approximately how many % of people are right-handed?
  - 80%
  - 75%
  - **90%**
  - 99%
2. Research shows that handedness is not based just on genetics because
  - Nothing is based just on genetics
  - Siblings do not always have the same dominant hand
  - **Scientists cannot find any genes that determine handedness across unrelated people**
  - None of the above
3. What is the Wada test?
  - **A way for doctors to determine which side of the brain contains language functions in a patient**
  - A way for government to stress-test financial systems
  - A way for doctors to examine possible brain atrophy
  - A way to determine whether a child is intellectually-gifted

## Footnotes

1 Note that these findings differ from interactions between WMC and math anxiety (see Beilock & Ramirez, 2011).

2 Note that Johns et al. referred to their intervention as a “teaching intervention.” We opted for the term “instructional intervention” because we thought a “teaching intervention” might lead some to believe that participants were asked to *teach someone else*, rather than *being taught something*.

3 The effect size is from the high anxiety students (based on a median split) from Ramirez and Beilock’s (2011) Experiments 3 and 4, during which students completed a biology exam. We opted to use these experiments because they were conducted in a classroom setting whereas Ramirez and Beilock’s other experiments were completed in a laboratory. The power analysis was conducted by dividing our sample size by 2 to match the median split design from Ramirez and Beilock (2011).

4 Students could earn the same amount of extra credit by reading a research article and completing a short quiz.

5 Note that the S-TAI does not include the same subscales (i.e., Worry, Emotionality) as the T-TAI.

6 Due to loss of data, 14 students’ writing tasks from the first semester were not available to analyze in the LIWC software.

7 Results did not differ when the data from Exams 4 and 5 were included.

8 To our knowledge, Bayes factors cannot be calculated for *z-*tests.

9 One might wonder why the correlations between the T-TAI and exam scores were substantially weaker when considering the data for Exams 2 and 3 than Exam 1. This is to be expected because the strong correlation between T-TAI and exam scores was produced based on the ranked scores of Exam 1. Therefore, the weaker correlations between T-TAI and exam scores under the control-writing and expressive-writing conditions relative to the Exam 1 baseline should not be taken as evidence that either control or expressive writing served to weaken the impact of test anxiety on exam performance.

10 The effect size is estimated by calculating the difference in effect size of gender between participants in the math test condition (i.e., participants under stereotype threat) and the teaching intervention condition (i.e., participants read a brief paragraph about stereotype threat). Johns et al. (2005) did not report the effect size for participants in the teaching intervention condition, so we used their reported means and standard errors from the figure to estimate the effect size in this condition based on the assumption that they had the same number of participants across the math test and teaching intervention condition (sample size was needed to estimate the standard deviation).

11 Results did not differ when data from Exam 3 and 4 were included.
